# *Plasmodium vivax* Parasite Load Is Associated With Histopathology in *Saimiri boliviensis* With Findings Comparable to *P vivax* Pathogenesis in Humans

**DOI:** 10.1093/ofid/ofz021

**Published:** 2019-01-19

**Authors:** Mariko S Peterson, Chester J Joyner, Regina J Cordy, Jorge L Salinas, Deepa Machiah, Stacey A Lapp, MaHPIC Consortium, Esmeralda V S Meyer, Sanjeev Gumber, Mary R Galinski

**Affiliations:** 1 Emory Vaccine Center, Yerkes National Primate Research Center; 2 Division of Infectious Diseases, Department of Medicine, School of Medicine; 3 Division of Pathology, Yerkes National Primate Research Center; 4 Department of Pathology and Laboratory Medicine, School of Medicine, Emory University, Atlanta, Georgia

**Keywords:** malaria, animal models, nonhuman primates, parasite tissue load, infectious diseases

## Abstract

**Background:**

*Plasmodium vivax* can cause severe malaria with multisystem organ dysfunction and death. Clinical reports suggest that parasite accumulation in tissues may contribute to pathogenesis and disease severity, but direct evidence is scarce.

**Methods:**

We present quantitative parasitological and histopathological analyses of tissue sections from a cohort of naive, mostly splenectomized *Saimiri boliviensis* infected with *P vivax* to define the relationship of tissue parasite load and histopathology.

**Results:**

The lung, liver, and kidney showed the most tissue injury, with pathological presentations similar to observations reported from autopsies. Parasite loads correlated with the degree of histopathologic changes in the lung and liver tissues. In contrast, kidney damage was not associated directly with parasite load but with the presence of hemozoin, an inflammatory parasite byproduct.

**Conclusions:**

This analysis supports the use of the *S boliviensis* infection model for performing detailed histopathological studies to better understand and potentially design interventions to treat serious clinical manifestations caused by *P vivax*.

*Plasmodium vivax* is the most widespread malaria parasite and causes an estimated 15.8 million clinical cases annually [1]. Although *P vivax* can cause serious and fatal disease [2], the mechanisms that underpin these complications remain poorly defined [3, 4].

Several studies have explored the association between the *P vivax* parasite tissue load and disease severity. Clinical studies have correlated plasma parasite biomass markers to systemic inflammatory markers [5] and intravascular accumulation of immune cells or parasites to lung injury [6]. Autopsy analyses have demonstrated the presence of parasites in histopathological sections [2], and in vitro adhesion of *P vivax*-infected red blood cells (iRBCs) to lung endothelial cells suggests that a cytoadhesion mechanism may be functioning in the lungs [7–9]. However, none of these studies have directly examined how histopathology relates to tissue parasite load.

Pathology studies with human cases can be limited by confounding factors such as coinfections, chronic diseases, and lack of information on exposure status or duration of infection. Nonhuman primate (NHP) models can be used to overcome these limitations and expand knowledge on *P vivax* pathogenesis, through the direct study of *P vivax* in New World monkeys [10, 11].

In this study, we demonstrate that histopathology is directly associated with parasite prevalence and hemozoin deposition in specific tissues. We scored the histopathology observed in organ tissue sections from a cohort of *Saimiri boliviensis* that were experimentally infected with *P vivax* (Brazil VII strain) [12] and quantified the parasite load in each tissue type to assess the relationship of parasite load and the severity of histopathology. Pathology was mainly observed in the lungs, liver, and kidneys and determined to be generalizable to findings from malaria autopsy cases [2, 13].

## METHODS

### Tissue Acquisitions From *Saimiri boliviensis* Infected With *Plasmodium vivax*

Tissues were collected at necropsy from 7 *S boliviensis* that were infected with *P vivax* Brazil VII strain iRBCs [12] for multiomic studies, as described in detail elsewhere [14, 15]. Healthy, malaria-naive squirrel monkeys (*S boliviensis*, 6 males, 1 female) were acquired from the Michale E. Keeling Center for Comparative Medicine and Research at the University of Texas-MD Anderson Cancer Center and transferred to the Yerkes National Primate Research Center (YNPRC), an AAALAC International-accredited facility. Male monkeys were preferably selected to eliminate confounding variables such as anemia stemming from menstruation. Six monkeys were splenectomized before infection to ensure highest parasitemias, and 1 was left intact. Appropriate enrichment activities were provided and before being infected, the animals received positive reinforcement training to perform leg-prick procedures to attain capillary blood samples for making smears. Veterinary and behavior experts monitored the animals daily for signs of disease and distress. All experimental plans were approved by Emory University’s Institutional Animal Care and Use Committee (IACUC).

To initiate infections, approximately 1 × 10^7^ cryopreserved iRBCs were thawed and injected intravenously into 1 *S boliviensis*. The remaining monkeys were serially inoculated by transferring 0.5 mL parasitized blood from infected monkeys when their parasitemia was approximately 1%. Parasitemias were monitored daily by quantifying the number of iRBCs out of 1000 total RBCs or by the Earle and Perez method [16] in Giemsa-stained thin and thick films, respectively. As parasitemias peaked and at necropsy, peripheral blood was collected, and aliquots were cryopreserved for future use [14, 15]. Each animal was euthanized according to IACUC-approved guidelines. Infected tissue samples were collected from selected organs as noted in the results. Normal tissue sections were obtained for comparison from the YNPRC archival bank of NHP specimens.

### Histopathology, Pathology Scoring, and Parasite Quantification

Tissue samples were fixed in 10% neutral buffered formalin, paraffin embedded, sectioned at 4 μm, and stained with hematoxylin and eosin (H&E) [17]. Where indicated, sections were also stained with Masson’s trichrome or Perl’s stain [17]. Sections were blinded for pathological and parasitological analysis. The organs were scored in 11 categories: inflammation, edema, necrosis, hemorrhage, hyperplasia, fibrosis, vasculitis, hypoplasia, degeneration, hemosiderosis, and others. Within each of these categories, the scores were determined on a range from 0 to 4, with 0 representing normal tissue, 1 minimal damage, 2 mild damage, 3 moderate damage, and 4 severe damage. Scores were then summed to obtain a total pathology score for each organ for every monkey. Parasites were quantified by counting the total number of parasites observed (mature trophozoites and schizonts) in 60 consecutive, ×1000 light microscopic fields. In total, 319 sections were quantified for parasite load and examined histologically. These included multiple sections for every organ to ensure representative coverage of the pathology and parasite load of each organ.

### Immunohistochemistry

Immunohistochemical staining of lung, liver, and kidney sections was performed using a biotin-free polymer system. The sections were deparaffinized in xylene, rehydrated in graded series of ethanol, and rinsed with distilled water. Antigen retrieval was performed by immersing sections in DIVA Decloaker (Biocare Medical) at 125°C for 30 seconds in a steam pressure decloaking chamber (Biocare Medical) followed by blocking for 10 minutes with SNIPER Reagent (Biocare Medical). The sections were incubated with mouse anti-human CD163 (clone 10D6; Abcam), mouse anti-human thyroid transcription factor 1 (TTF-1) (clone 8G7G3/1; Dako), or mouse anti-*Plasmodium* lactate dehydrogenase (pLDH) (clone 19; MyBioSource) overnight at 4°C followed by a detection polymer system (MACH 2 [Biocare Medical], or in the case of anti-TTF1 staining, EnVision+ System-HRP Labeled Polymer [Dako]). Antibody labeling was visualized by alkaline phosphatase chromogen development (Warp Red Chromogen Kit; Biocare Medical), or for anti-TTF1 staining, immunoperoxidase with 3-3’-diaminobenzidine. Nuclei were counter stained using Mayer’s hematoxylin (Vector Laboratories).

### Statistical Analysis

The Fisher’s exact test, multiple linear regression, and Spearman correlations were calculated to determine relationships between pathology score and tissue parasite load. The Tukey post hoc honest significant difference (HSD) comparisons test was used to determine significant differences between parasite load and histopathology score groups. All statistical analyses were produced using R Studio version 1.1.383, under R version 3.4.3 GUI version 1.70.

## RESULTS

### Parasite Kinetics of *Plasmodium vivax* Brazil VII Infection in *Saimiri boliviensis*

Parasitemia was monitored daily after inoculation of the monkeys (Supplemental Figure 1) [18] (mean maximum parasitemia was 70 572 parasites/μL with a range of 55 000–90 000 parasites/μL). Mean parasitemia at necropsy was 61 572 parasites/μL, and the range was 25 000–90 000 parasites/μL. The animals were euthanized after reaching a sustained parasitemia close to 1% and immediately necropsied for pathology analyses. The mean duration of infection was 15.71 days (with a range of 10–24 days). Infection parameters are summarized in Supplemental Table 2.

### *Plasmodium vivax* Brazil VII Infection of *Saimiri boliviensis* Causes Lung, Liver, and Kidney Tissue Damage

**Figure 1.**
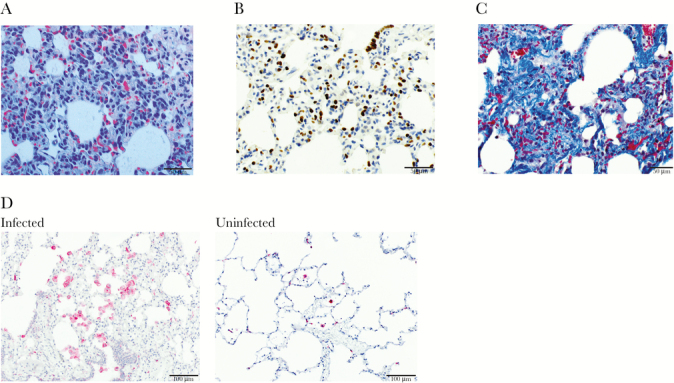
Lung tissue sections with representative micrographs showing histopathology. (A) Marked type-II pneumocyte hyperplasia alveolar wall thickening and inflammation in a splenectomized monkey, and hemozoin-laden cell infiltration in a hematoxylin and eosin stain-stained section under polarized light. Scale bar = 50 μm. (B) anti-thyroid transcription factor 1 (TTF1) immunohistochemical staining highlights type II pneumocyte hyperplasia in a splenectomized monkey. Type II pneumocytes are indicated by dark brown labeling. Scale bar = 50 μm. (C) Extensive interstitial collagen deposition in alveolar wall stained blue with Masson’s trichrome in the spleen-intact monkey. Scale bar = 50 μm. (D) Numerous immunohistochemically labeled CD163^+^ cells (fuschin red) infiltrate the alveoli and alveolar walls in the spleen-intact monkey (left) relative to few CD163^+^ cells in the lungs of an uninfected *Saimiri boliviensis* monkey (right). Scale bar = 100 μm.

**Figure 2.**
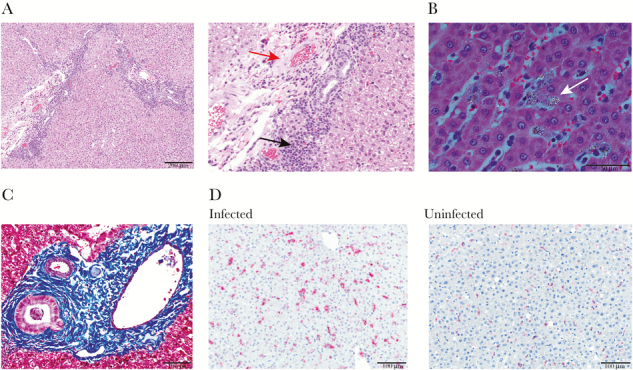
Liver tissue sections of splenectomized monkeys with representative micrographs showing histopathology. (A) Hematoxylin and eosin (H&E)-stained section shows small amounts of collagen deposition and 2 foci of mononuclear periportal infiltrate (left). Scale bar = 200 μm. A zoomed-in image at the same magnification is shown to highlight the collagen deposition (red arrow) and mononuclear infiltrate (black arrow). (B) The H&E-stained section viewed with polarized light shows hemozoin-laden macrophages highlighted by white birefringence with sinusoidal congestion (white arrow). Scale bar = 50 μm. (C) Masson’s trichrome-stained section with collagen deposition in the periportal region shown by deep blue staining. Scale bar = 100 μm. (D) Immunohistochemically stained section showing numerous CD163^+^-stained cells (fuschin red) and counter stained with hematoxylin in the hepatic parenchyma (left) relative to uninfected *Saimiri boliviensis* liver tissue (right). Scale bar = 100 μm.

**Figure 3.**
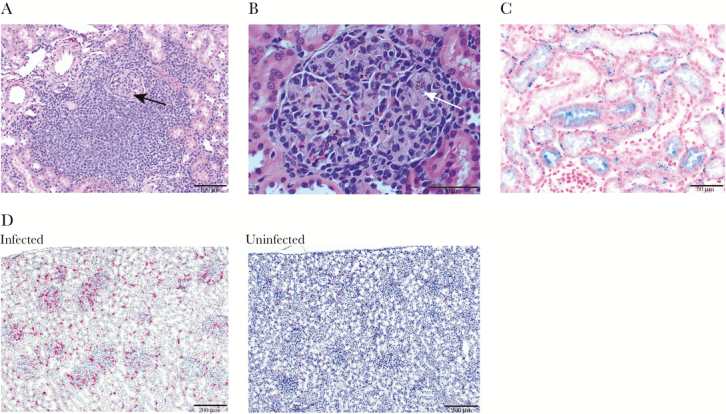
Kidney tissue sections of splenectomized monkeys with representative micrographs showing histopathology. (A) A Hematoxylin and eosin (H&E)-stained section of renal cortex showing massive mononuclear infiltrate surrounding a glomerulus (black arrow). Scale bar = 100 μm. (B) The H&E section with polarized light showing a hypercellular glomerulus with hemozoin-laden macrophage infiltrate (white arrow). Scale bar = 50 μm. (C) Positive Perl’s stain in the tubules showing hemosiderin deposits (blue). Counter stain is eosin (pink). Scale bar = 50 μm. (D) Marked CD163^+^ cell infiltration in the glomeruli and renal cortex (fuschin red) and counter stained with hematoxylin in this immunohistochemical-stained section, relative to an uninfected *Saimiri boliviensis* kidney section (right). Scale bar = 200 μm.

Lung, liver, kidney, brain, gastrointestinal tract, gonads, heart, adrenal gland, lymph nodes, bone marrow, and spleen (from the 1 spleen-intact monkey) were acquired at necropsy for histopathology analyses. Brain, gastrointestinal tract, gonads, heart, adrenal gland, and lymph node sections did not reveal major pathological lesions specific to malaria. However, the lungs, liver, and kidneys exhibited significant histopathological damage (Supplemental Table 2, Figures 1–3) similar to that observed in human malaria cases [2, 19]. In addition, H&E-stained sections of the bone marrow revealed hypercellularity in all monkeys.

All monkeys had mild to moderate pulmonary edema. Multifocal areas of alveolar wall thickening and inflammation were observed in the lung tissues consistent with interstitial pneumonia (Figure 1A). Type II cell hyperplasia, indicative of lung injury, was demonstrated by increased numbers of cells labeled with anti-TTF-1 (Figure 1B). Interstitial collagen deposition was observed in the lung sections from 2 monkeys (Figure 1C). The tissues from 4 monkeys exhibited focal hemorrhage. Birefringent pigment consistent with hemozoin was diffusely observed in CD163^+^ macrophages and infected erythrocytes [20]. Infiltration of CD163^+^ cells was also observed in the alveolar wall and the alveoli, and these were increased relative to normal *Saimiri* lung tissue (Figure 1D).

Diffuse hepatocellular vacuolar degeneration was observed in all monkeys (Figure 2A). The periportal areas were infiltrated by moderate numbers of mononuclear cells (Figure 2A). Pigment-laden macrophages and pigmented parasites were diffusely distributed in the sinusoids throughout the liver. The pigment was birefringent under polarized light (Figure 2B). In 2 individuals, the portal areas were occasionally expanded by a small to moderate amount of collagen (Figure 2A), also shown highlighted by Masson’s trichrome (Figure 2C). In addition, the liver parenchyma had an increased number of pigment-laden CD163^+^ macrophages with morphology consistent with Kupffer cells compared with normal *Saimiri* liver tissue (Figure 2D). Overall, inflammation and fibrosis were localized in the perivascular spaces, including sinusoidal and periportal regions, suggesting liver injury originating from the presence of circulating parasites.

The kidneys showed multifocal cortical interstitial mononuclear infiltrates, especially in the periglomerular regions, consistent with nephritis (Figure 3A). The tubules were eosinophilic and swollen, with hyaline casts. Each infected monkey had enlarged, hypercellular glomeruli, infiltrated with hemozoin-laden CD163^+^ macrophages, relative to normal *Saimiri* glomeruli (Figure 3B and D). To differentiate hemosiderin, which can result from erythrocyte lysis, and parasite hemozoin, Perl’s stain was used, which reacts with hemosiderin, coloring it blue. This stain does not react with hemozoin, leaving it black. It is interesting to note that Perl’s stain revealed localization of pigment consistent with hemosiderin in the glomeruli and stained granules in the tubules blue, consistent with hemosiderin secretion (Figure 3C).

**Figure 4.**
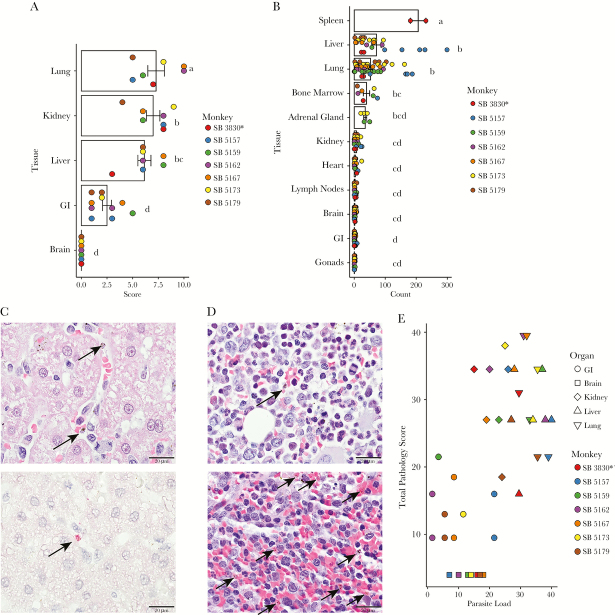
Histopathological and parasitological tissue analysis, in the organs of 7-infected *Saimiri boliviensis* with asterisk indicating spleen-intact animal. (A) Histopathological tissue scores: the means, data points, and standard errors are reported, with the height of the bar showing the mean, the dots indicating individual data points, and the error bars showing the standard error of the mean. Statistical significance as determined by the Tukey honest significant difference test is indicated by letters above the bars. Each data point represents the whole organ score obtained from examination of hematoxylin and eosin (H&E)-stained tissue sections. (B) Quantification of parasite load in the organs. Three hundred nineteen sections were examined. Each data point represents the total number of parasites in 60 consecutive oil-immersion (×1000) fields in a single H&E-stained section. (C, top) Mature parasites are readily identifiable in H&E-stained sections (black arrows). Scale bar = 20 μm. (C, bottom) Select sections were stained with antiparasite lactate dehydrogenase antibody (fuschin red) and counterstained with hematoxylin as an alternative means to show and confirm parasite presence (black arrow). Scale bar = 20 μm. (D) Representative H&E-stained sections of bone marrow from a splenectomized animal (top), and the spleen from the intact animal (bottom) indicating the distribution of parasites (black arrows). (E) Histopathological score versus organ parasite load. Scatter plot of the rank of the sum of the parasite counts in 60 consecutive oil-immersion (×1000) fields in all sections of a given organ in a given monkey plotted against the rank of the total pathology score. Only tissues with scores were included.

To compare the extent of histopathological changes, the tissues from each infected *S boliviensis* were scored, on a scale from 0 to 4, in 11 criteria, and these criteria were summed to obtain a total pathology score for each organ. Mean total pathology scores (Figure 4A) for the organs of 7 monkeys were highest for lung (mean = 7.29, standard error = ±0.81), kidney (mean = 7.00, standard error = ±0.65), and liver (mean = 6.14, standard error = ±0.63). The gastrointestinal tissues, colon, and stomach (mean = 2.67, standard error = ±0.67 and mean = 2.33, standard error = ± 0.61, respectively) had low scores. The brain was normal (mean = 0, standard error = ±0). The mean score across all tissues was 5, standard error = ±0.50, and the median was 4.33. Lymph nodes, gonads, heart, spleen, and bone marrow were not scored because they showed no major histopathological lesions. Statistically significant differences between organ scores were assessed using Tukey’s HSD post hoc pairwise comparison test. The mean pathology scores of kidney, liver, and lung, were significantly higher than brain, stomach, and colon (Supplemental Table 3).

### Tissue Damage Is Associated With Parasite Prevalence in Specific Organs

To test whether *P vivax* parasite load correlated with organ pathology, parasite counts were obtained from sections of 10 organs, plus the spleen from the intact animal (Figure 4B). In total, parasites were counted in 319 sections. Trophozoites, schizonts, and gametocytes can be readily visualized in H&E-stained histological sections (Figure 4C) [21], and select sections were also stained with anti-pLDH antibody as a confirmation method (Figure 4C) [10, 22]. The mean number of parasites across all sections was 17.61 per ×1000 high-power field (HPF), with a standard error of 2.24. As expected due to high erythrocyte density in the red pulp (Figure 4D), the spleen sections contained the most parasites per HPF (the mean between 2 sections from the nonsplenectomized animal was 206, with a standard error of 25). The liver (mean = 71.56, standard error = ±15.11), lungs (mean = 52.20, standard error = ±7.13), bone marrow (mean = 39.43, standard error = ±10.08) (Figure 4D), and adrenal glands (mean = 35.2, standard error = ±4.97) also carried significant parasite loads. The gastrointestinal organs (mean = 0.92, standard error = ±0.18), brain (mean = 1.95, standard error = ±0.32), heart (mean = 4.05, standard error = ±1.2), and kidneys (mean = 7.35, standard error = ±1.27) exhibited low parasite loads. The gonads and lymph nodes also exhibited a paucity of parasites (mean = 0.8, standard error = ±0.18 and mean = 2.97, standard error = ±0.59, respectively). Parasite counts were analyzed for significant differences using the Tukey’s HSD post hoc pairwise comparisons tests (Supplemental Table 4). The spleen tissue section counts were significantly higher compared with all other organs. The liver and lung parasite counts were not significantly different from one another, nor from the bone marrow and adrenal gland counts. However, they were significantly higher than the parasite counts from kidney, heart, lymph node, brain, gastrointestinal tract, and gonad sections.

To test the association between parasite load and histopathological scores, the parasite count data from individual sections were averaged to obtain whole organ counts for each monkey, and these were divided into high parasite load (greater than the mean of the organ parasite averages, 23) and low parasite load (less than 23) categories. Only organs for which there were scores available were considered. Organ histopathological scores were divided into high score (greater than the mean score, 5) and low score (less than 5). The resultant 2 × 2 contingency table is presented in Supplemental Table 5. Organs with high pathology scores were 22.09 times more likely to have a high parasite load than a low parasite load, with a *P* = .00057.

To test the correlation between histopathological score and parasite load, multiple linear regression was performed. The parasite count was weakly, but significantly positively linearly associated with pathology score, with an adjusted R^2^ = 0.105 and *P* = .0235 (Supplemental Table 6). However, once organ was added as an effect in the regression model, the trend became strongly linear (R^2^ = 0.7505, *P* < .00001), with the organ effect being so strong that the contribution of parasite count to pathology score was no longer significant. Multicollinearity and interaction between count and organ were ruled out. Additional parameters that might affect histopathology including the maximum parasitemia (defined as the maximum parasitemia reached in parasites/μL), days at parasitemia equal to or greater than 50 000 parasites/μL, the proportion of days with parasitemias equal to or greater than 50 000 parasites/μL, the duration of the infection, and the parasitemia at the time of necropsy were also considered and tested using a hierarchical multiple linear regression modeling approach. None of these parameters contributed significantly to the model.

As the regression analysis demonstrates, pathology score and parasite count do not have a linear relationship. To test the association between pathology score and parasite count, Spearman correlation coefficients were calculated (Supplemental Table 7). Here, pathology score showed a significantly and moderately strong positive association with parasite count, with Spearman’s ρ = 0.6034, and an adjusted *P* = .0002 (ranked values plotted in Figure 4E). Maximum parasitemia, days at parasitemia greater than or equal to 50 000 parasites/μL, proportion of days at or greater than 50 000 parasites/μL, duration of infection, and parasitemia at the time of necropsy were also tested, and none of the resultant ρ values were found to be significant. *P* values were corrected using Bonferroni’s post hoc correction to account for multiple tests.

## DISCUSSION

This quantitative analysis of tissues from longitudinal infections of *P vivax* (Brazil VII) in a cohort of naive *S boliviensis* has enabled the discovery of specific relationships between parasite load and histopathology. Previous work showed that a reduction of the proportion of circulating schizonts compared with rings and trophozoites in clinical cases is associated with increased adherence of the more mature iRBCs to endothelial cell lines [9], potentially mediated by membrane-associated variant interspersed repeat (VIR) proteins [7]. In addition, gas exchange studies have pointed to parasite or immune cell accumulation in lung vasculature as a possible mechanism for respiratory distress in patients [6]. Recent retrospective tissue analyses of *P vivax* (Salvador-I strain) infections of nonnaive *Aotus nancymae* and *S boliviensis* monkeys highlighted enrichment of parasites in the bone marrow, liver, and lungs, providing further evidence of the accumulation of *P vivax* iRBCs in tissues [10].

Important pathological findings from *P vivax* clinical cases have been replicated here in the *S boliviensis* model, particularly in the lungs, liver, and kidneys. Mononuclear infiltrates were evident in tissue sections from lungs, periportal areas of the liver, and kidneys. This is consistent with autopsy assessments from human *P vivax* and *P falciparum* cases [2, 13, 19, 23] and necropsy analysis from a severe infection of the *P vivax* model organism, *Plasmodium cynomolgi* in a rhesus macaque [24]. More macrophages were observed in the lungs, liver, and kidneys, compared with uninfected individuals. Alveolar wall thickening with macrophage infiltration, type II pneumocyte hyperplasia and collagen deposition, and histological evidence of pulmonary edema were all evident in the *S boliviensis* tissues. Respiratory complications in vivax malaria have been well documented [13, 25–27] and associated with significant mortality in humans [28], and *P vivax* iRBCs have been visualized in the lungs of autopsy cases [2]. Periportal inflammation, sinusoidal congestion, and Kupffer cell hyperplasia have been demonstrated here and with severe *P falciparum* malaria [29]. Hemozoin-laden CD163^+^ macrophages were identified in the kidneys (Figure 4B) and noted with *P falciparum*-associated kidney damage [23]. Finally, a significant number of *P vivax* iRBCs were observed in the bone marrow. This is consistent with studies noting enrichment of *P vivax* and *P falciparum* iRBCs and hemozoin in the bone marrow, especially gametocytes, in support of this tissue being a niche for parasite persistence to ensure transmission [10, 30].

The linear regression analysis indicated that organ-specific tissue injury was attributed to the presence of parasites. The lungs and liver exhibited high parasite loads and corresponding high pathology scores. On the other hand, the kidneys had low parasite counts but significant damage. We speculate that this represents an example of organ-specific injury arising from systemic or local inflammation due to the presence of parasite byproducts; namely, hemozoin. It is striking that almost every glomerulus contained hemozoin-pigmented macrophages. Hemozoin is an immunomodulatory insoluble crystalline metabolite of heme detoxification [31], which has been implicated in kidney damage with *Plasmodium berghei* infection [32], chronic inflammation after *Plasmodium yoelli* and *Plasmodium chabaudi* blood-stage infections [33], and dyserythropoiesis in *Plasmodium falciparum* malaria [34]. In addition, Perl’s stain confirmed the presence of hemosiderin in the renal tubules, consistent with RBC destruction. Hemosiderin is a byproduct of erythrocyte lysis, and studies have shown that most RBCs destroyed during malaria are uninfected [35, 36]. Hemosiderin deposition as a sentinel to free hemoglobin and heme-mediated tissue injury during malaria has been associated with acute kidney injury [37].

The spleen is a critical organ for malarial immunity [38–40]. More importantly, the pathology scores overall across the tissues from the spleen-intact animal were comparable to those from the splenectomized animals, suggesting that splenectomies intended to increase parasitemias may not be needed to study pathology. That is, next to the spleen, most parasites from this animal were similarly found in the liver, lung, and bone marrow. Another recent NHP study involving *P vivax*-infected common squirrel monkeys (*Saimiri sciureus*) and night monkeys (*Aotus lemurinus lemurinus*), which quantified parasites from organ crushes and blood films, suggested that *P vivax* parasites preferentially distribute to the spleen, and then to secondary sites [11]. Our observations are consistent with this interpretation. This increased distribution to secondary sites in the absence of a spleen may also explain the relatively large parasite load observed in the bone marrow by Obaldia et al [10].

Parasite biomass has been associated with increased disease severity in clinical studies; however, these findings in humans failed to find an association between parasite biomass and soluble endothelial activation markers systemically [5]. The authors proposed a significant parasite reservoir in organs where the microvasculature is not lined with endothelium, such as the bone marrow and spleen [5]. Our observations of significant numbers of parasites in the spleen of the intact monkey, primarily concentrated in the red pulp, and reduced numbers of parasites in other organs relative to the splenectomized individuals support this hypothesis. Parasite or parasite byproduct presence in specific organs, such as the lung, kidney, or liver, may cause local inflammation, tissue damage, and disease manifestations, as we observed, but may not result in systemic endothelial activation.

This study, which directly associates organ parasite load with histopathology using an NHP model of *P vivax* infection, is not without limitation. The animals were splenectomized to ensure higher peripheral parasitemias for the collection of iRBCs. The experimental removal of the spleens could raise questions on the translatability of this model to humans. However, the data from the single spleen-intact animal used for comparisons here suggests that histopathology will be similar irrespective of the presence or absence of a spleen. Notably, this conclusion must be interpreted with caution, however, because only a single spleen-intact animal was included here. Nevertheless, our data suggest that spleen-intact animals can be used for future studies with this parasite strain. Finally, complete blood counts and blood chemistries were not available for analysis here due to the retrospective nature of the study. This limitation makes it difficult to correlate the histopathology with systemic changes indicative of disease such as decreases in hemoglobin, increases in liver enzymes, indicators of lung function, or disruption of kidney function. Future prospective studies should include these measurements to determine how the histopathological changes may influence disease presentation.

## CONCLUSIONS

In summary, this quantitative study has demonstrated the accumulation of *P vivax* iRBCs in spleen, lung, liver, bone marrow, and adrenal gland tissues and a paucity of iRBCs in highly perfused organs including the gastrointestinal tract, heart, kidney, and brain. Histopathology was most notable in the lung, liver, and kidney. Moreover, the accumulation of iRBCs in tissues is positively associated with tissue damage, with multiple linear regression models suggesting a strong effect for tissue-specific histopathological changes. However, as shown for the kidney, disease processes can be attributed to parasite byproducts. Future research using this animal model could be warranted to study tissue-specific *P vivax* pathological manifestations, the relationship between parasite biomass, systemic immune activation and pathology, and to test interventions.

## Supplementary Data

Supplementary materials are available at *Open Forum Infectious Diseases* online. Consisting of data provided by the authors to benefit the reader, the posted materials are not copyedited and are the sole responsibility of the authors, so questions or comments should be addressed to the corresponding author.

ofz021_suppl_supplementary_table_1

ofz021_suppl_supplementary_table_2

ofz021_suppl_supplementary_table_3

ofz021_suppl_supplementary_table_4

ofz021_suppl_supplementary_table_5

ofz021_suppl_supplementary_table_6

ofz021_suppl_supplementary_table_7

ofz021_suppl_supplementary_fig_1

## Notes

Presented in part: 6th International Conference on *Plasmodium vivax* Research, Manaus, Brazil, June 11–14, 2017, abstract number 63215.
