# Supplementary material for: Plasmodium vivax Parasite Load Is Associated With Histopathology in Saimiri boliviensis With Findings Comparable to P vivax Pathogenesis in Humans
Source: Open Forum Infect Dis. 2019 Jan 19;6(3):ofz021. doi: 10.1093/ofid/ofz021 (PMC6436601; doi:10.1093/ofid/ofz021)
Supplement: ofz021_suppl_supplementary_table_1 [file ofz021_suppl_supplementary_table_1.docx]

**Supplemental Table 1: Infection Parameter Summary**

| Parameter | Mean (Range) |
| --- | --- |
| Age of monkey (years) | **5** (4-11) |
| Maximum parasitemia (parasites/μl) | **70,572** (55,000-90,000) |
| Duration of infection (days) | **15.71** (10-24) |
| Days with parasitemia > 50,000 parasites/μl (days) | **3.57** (1-8) |
| Proportion of days of infection with parasitemia > 50,000 parasites/μl | **0.231** (0.05-0.364) |
| Parasitemia at necropsy (parasites/ul) | **61,572** (25,000-90,000) |

**Supplemental Table 1:** Review of experimental demographics and parasite kinetics. Maximum parasitemia refers to the total parasitemia above 50,000 parasites/μl infected RBCs out of total RBCs. Proportion of infection with parasitemia 50,000 parasites/μl is defined as the total number of parasitemic days exceeding 50,000 parasites/μl divided by the total number of days infected. Average parasitemias were rounded up to the first whole number.
