# Supplementary material for: Plasmodium vivax Parasite Load Is Associated With Histopathology in Saimiri boliviensis With Findings Comparable to P vivax Pathogenesis in Humans
Source: Open Forum Infect Dis. 2019 Jan 19;6(3):ofz021. doi: 10.1093/ofid/ofz021 (PMC6436601; doi:10.1093/ofid/ofz021)
Supplement: ofz021_suppl_supplementary_table_2 [file ofz021_suppl_supplementary_table_2.docx]

| **Supplemental Table 2: Summary of Histopathology by Tissue for Each Monkey** | | | | | | | |
| --- | --- | --- | --- | --- | --- | --- | --- |
| Histopathology | SB 5173 | SB 5159 | SB 5157 | SB 5162 | SB 5167 | SB 5179 | SB 3830* |
| **Bone Marrow** |  |  |  |  |  |  |  |
| Hypercellularity | X | X | X | X | X | X | X |
| **Lungs** |  |  |  |  |  |  |  |
| Pulmonary edema | X | X | X | X | X | X | X |
| Alveolar wall thickening | X | X | X | X | X | X | X |
| Type II hyperplasia | X | X | X | X | X | X | X |
| Fibrosis | O | O | O | X | O | O | X |
| Alveolar and alveolar wall infiltration | X | O | X | X | X | X | X |
| Hemorrhage | O | X | O | X | X | X | O |
| **Liver** |  |  |  |  |  |  |  |
| Vacuolar degeneration | X | X | X | X | X | X | X |
| Periportal infiltrate | X | X | X | X | X | X | X |
| Sinusoidal infiltrate | X | X | X | X | X | X | X |
| Kupffer cell hyperplasia | X | X | X | X | X | X | X |
| Fibrosis | O | X | O | O | X | O | O |
| **Kidney** |  |  |  |  |  |  |  |
| Nephritis | X | X | X | X | X | X | X |
| Tubular degeneration | X | X | X | X | X | O | X |
| Glomerular hypercellularity | X | X | X | X | X | X | X |
| Hemorrhage | O | O | X | O | O | O | O |

**Supplemental Table 2:** Summary of histopathological findings. The presence (X) or absence (O) of major histopathological findings in the relevant organ systems by monkey are shown.
