# Supplementary material for: Plasmodium vivax Parasite Load Is Associated With Histopathology in Saimiri boliviensis With Findings Comparable to P vivax Pathogenesis in Humans
Source: Open Forum Infect Dis. 2019 Jan 19;6(3):ofz021. doi: 10.1093/ofid/ofz021 (PMC6436601; doi:10.1093/ofid/ofz021)
Supplement: ofz021_suppl_supplementary_table_3 [file ofz021_suppl_supplementary_table_3.docx]

| **Supplemental Table 3: Tukey HSD Post-Hoc Pairwise Comparison of Histopathology Scores** | | | | | | |
| --- | --- | --- | --- | --- | --- | --- |
| Organ 1 (I) | Organ 2 (J) | Mean Difference (I-J) | Adjusted P-value | Significance | LB | UB |
| Brain | Colon | 2.667 | 0.0524 | NS | -0.187 | 5.352 |
|  | Kidney | 7.000 | 0.0000 | **** | 4.420 | 9.580 |
|  | Liver | 6.143 | 0.0000 | **** | 3.563 | 8.723 |
|  | Lung | 7.286 | 0.0000 | **** | 4.706 | 9.866 |
|  | Stomach | 2.333 | 0.1192 | NS | -0.352 | 5.019 |
| Colon | Kidney | 4.333 | 0.0033 | ** | 1.648 | 7.019 |
|  | Liver | 3.476 | 0.0052 | ** | 0.791 | 6.162 |
|  | Lung | 4.619 | 0.0001 | *** | 1.933 | 7.304 |
|  | Stomach | -0.333 | 0.9991 | NS | -3.120 | 2.453 |
| Kidney | Liver | -0.857 | 0.9138 | NS | -3.437 | 1.722 |
|  | Lung | -0.286 | 0.0001 | *** | -2.294 | 2.866 |
|  | Stomach | -4.667 | 0.7627 | NS | -7.352 | -1.981 |
| Lung | Liver | 1.143 | 0.0018 | ** | -1.437 | 3.723 |
| Stomach | Liver | -3.810 | 0.0018 | ** | -6.495 | -1.124 |
|  | Lung | -4.952 | 0.0000 | **** | -7.638 | -2.267 |
| **Supplemental Table 3:** Pairwise histology score comparison. The Tukey HSD post-hoc pairwise comparison results for organ scores are summarized. Mean difference is significant at α = 0.05; **< 0.005; *** < 0.0005; **** < 0.00005; NS = not significant. LB = Lower Bound, UB = Upper Bound in the 95% Confidence Interval. | | | | | | |
