# Supplementary material for: Plasmodium vivax Parasite Load Is Associated With Histopathology in Saimiri boliviensis With Findings Comparable to P vivax Pathogenesis in Humans
Source: Open Forum Infect Dis. 2019 Jan 19;6(3):ofz021. doi: 10.1093/ofid/ofz021 (PMC6436601; doi:10.1093/ofid/ofz021)
Supplement: ofz021_suppl_supplementary_table_4 [file ofz021_suppl_supplementary_table_4.docx]

| **Supplemental Table 4: Tukey HSD Post-Hoc Pairwise Comparison of Parasite Organ Load** | | | | | | |
| --- | --- | --- | --- | --- | --- | --- |
| Organ 1 (I) | Organ 2 (J) | Mean Difference (I-J) | Adjusted P-value | Significance | LB | UB |
| Adrenal Gland | Bone Marrow | -4.229 | 1.0000 | NS | -49.970 | 58.426 |
|  | Brain | 33.249 | 0.3329 | NS | -77.094 | 10.597 |
|  | GI | 33.280 | 0.2469 | NS | -76.833 | 8.274 |
|  | Heart | 31.152 | 0.5112 | NS | -77.211 | 14.907 |
|  | Kidney | 27.845 | 0.6320 | NS | -72.453 | 16.762 |
|  | Liver | -36.360 | 0.2530 | NS | -9.985 | 81.705 |
|  | Lung | -16.996 | 0.9739 | NS | -26.590 | 60.581 |
|  | Lymph Node | 32.330 | 0.4395 | NS | -78.003 | 13.342 |
|  | Reproductive Tract | 34.400 | 0.3114 | NS | -79.111 | 10.311 |
|  | Spleen | -170 | 0.0000 | **** | 248.241 | 93.359 |
| Bone Marrow | Brain | 37.477 | 0.0550 | NS | -75.330 | 0.376 |
|  | GI | 38.508 | 0.0276 | * | -74.857 | -2.159 |
|  | Heart | 35.381 | 0.1480 | NS | -75.778 | 5.016 |
|  | Kidney | 32.074 | 0.2114 | NS | -70.807 | 6.660 |
|  | Liver | -32.131 | 0.2367 | NS | -7.449 | 71.712 |
|  | Lung | -12.767 | 0.9906 | NS | -24.785 | 50.319 |
|  | Lymph Node | 36.559 | 0.1077 | NS | -76.514 | 3.396 |
|  | Reproductive Tract | 38.629 | 0.0528 | NS | -77.481 | 0.223 |
|  | Spleen | -166.571 | 0.0000 | **** | 92.358 | 240.785 |
| Brain | GI | 1.031 | 1.0000 | NS | -18.533 | 16.471 |
|  | Heart | -2.096 | 1.0000 | NS | -22.742 | 26.934 |
|  | Kidney | -5.403 | 0.9994 | NS | -16.627 | 27.434 |
|  | Liver | -69.609 | 0.0000 | **** | 46.121 | 93.096 |
| **Supplemental Table 4: Tukey HSD Post-Hoc Pairwise Comparison of Parasite Organ Load (Continued)** | | | | | | |
| Organ 1 (I) | Organ 2 (J) | Mean Difference (I-J) | Adjusted P-value | Significance | LB | UB |
|  | Lung | -50.244 | 0.0000 | **** | 30.364 | 70.124 |
|  | Lymph Nodes | -0.918 | 1.0000 | NS | -23.195 | 25.032 |
|  | Reproductive Tract | 1.151 | 1.0000 | NS | -23.389 | 21.087 |
|  | Spleen | -204.049 | 0.0000 | **** | 137.022 | 271.076 |
| GI | Heart | -3.127 | 1.0000 | NS | -19.352 | 25.607 |
|  | Kidney | -6.434 | 0.9920 | NS | -12.898 | 25.767 |
|  | Liver | -70.640 | 0.0000 | **** | 49.662 | 91.617 |
|  | Lung | -51.276 | 0.0000 | **** | 34.435 | 68.116 |
|  | Lymph Nodes | -1.949 | 1.0000 | NS | -19.727 | 23.625 |
|  | Reproductive Tract | 0.120 | 1.0000 | NS | -19.689 | 19.448 |
|  | Spleen | -205.080 | 0.0000 | **** | 138.890 | 271.269 |
| Heart | Kidney | -3.307 | 1.0000 | NS | -22.853 | 29.467 |
|  | Liver | -67.512 | 0.0000 | **** | 40.114 | 94.911 |
|  | Lung | -48.148 | 0.0000 | **** | 23.771 | 72.525 |
|  | Lymph Nodes | 1.178 | 1.0000 | NS | -29.115 | 26.759 |
|  | Reproductive Tract | 3.248 | 1.0000 | NS | -29.583 | 23.088 |
|  | Spleen | -201.952 | 0.0000 | **** | 133.457 | 270.448 |
| Kidney | Liver | -64.205 | 0.0000 | **** | 39.324 | 89.086 |
|  | Lung | -44.841 | 0.0000 | **** | 23.332 | 66.349 |
|  | Lymph Nodes | 4.485 | 1.0000 | NS | -29.958 | 20.987 |
|  | Reproductive Tract | 6.555 | 0.9982 | NS | -30.260 | 17.151 |
|  | Spleen | -198.645 | 0.0000 | **** | 131.117 | 266.173 |
| Liver | Lung | 19.364 | 0.1912 | NS | -42.363 | 3.634 |
| **Supplemental Table 4: Tukey HSD Post-Hoc Pairwise Comparison of Parasite Organ Load (Continued)** | | | | | | |
| Organ 1 (I) | Organ 2 (J) | Mean Difference (I-J) | Adjusted P-value | Significance | LB | UB |
|  | Lymph Nodes | 68.690 | 0.0000 | **** | -95.433 | -41.947 |
|  | Reproductive Tract | -70.760 | 0.0000 | **** | -95.825 | -41.695 |
|  | Spleen | -134.440 | 0.0000 | **** | 66.422 | 202.458 |
| Lung | Lymph Nodes | 49.326 | 0.0000 | **** | -72.964 | -25.688 |
|  | Reproductive Tract | 51.396 | 0.0000 | **** | -73.117 | -29.674 |
|  | Spleen | -153.804 | 0.0000 | **** | 86.947 | 220.662 |
| Lymph Nodes | Reproductive Tract | 2.070 | 1.0000 | NS | -27.723 | 23.583 |
|  | Spleen | -203.130 | 0.0000 | **** | 134.894 | 271.367 |
| Spleen | Reproductive Tract | -205.200 | 0.0000 | **** | 137.604 | 272.796 |
| **Supplemental Table 4:** Tukey HSD post-hoc pairwise comparison. Pairwise comparison of parasite counts is summarized. Mean difference is significant at α = 0.05; **< 0.005; *** < 0.0005; **** < 0.00005; NS = not significant. LB = Lower Bound, UB = Upper Bound in the 95% Confidence Interval. | | | | | | |
