# Supplementary material for: Plasmodium vivax Parasite Load Is Associated With Histopathology in Saimiri boliviensis With Findings Comparable to P vivax Pathogenesis in Humans
Source: Open Forum Infect Dis. 2019 Jan 19;6(3):ofz021. doi: 10.1093/ofid/ofz021 (PMC6436601; doi:10.1093/ofid/ofz021)
Supplement: ofz021_suppl_supplementary_table_5 [file ofz021_suppl_supplementary_table_5.docx]

| **Supplemental Table 5: Fisher Exact Test Contingency Table** | | |
| --- | --- | --- |
|  | High Parasite Burden (%) | Low Parasite Burden (%) |
| High Score | 12 (92%) | 9 (33%) |
| Low Score | 1 (8.3%) | 18 (67%) |
|  | | |

**Supplemental Table 5:** Fisher exact test. The Fisher Exact Test Contingency table and results are summarized. α = 0.05; P = 0.0005712; Odds ratio: 22.09.
