# Supplementary material for: Plasmodium vivax Parasite Load Is Associated With Histopathology in Saimiri boliviensis With Findings Comparable to P vivax Pathogenesis in Humans
Source: Open Forum Infect Dis. 2019 Jan 19;6(3):ofz021. doi: 10.1093/ofid/ofz021 (PMC6436601; doi:10.1093/ofid/ofz021)
Supplement: ofz021_suppl_supplementary_table_6 [file ofz021_suppl_supplementary_table_6.docx]

| **Supplemental Table 6: Linear Regression** | | | | | | | | | | |
| --- | --- | --- | --- | --- | --- | --- | --- | --- | --- | --- |
|  | Model 1 | | | |  | Model 2 | | | |  |
|  | Estimate | Std. Error | t value | P-value | Significance | Estimate | Std. Error | t value | P-value | Significance |
| Intercept | 3.676 | 0.549 | 6.699 | 0.0000 | **** | 2.509 | 0.458 | 5.482 | 0.0000 | **** |
| Count | 0.028 | 0.012 | 2.361 | 0.0235 | * | -0.008 | 0.009 | -0.858 | 0.3989 | NS |
| Organ |  |  |  |  |  |  |  |  |  |  |
| *Brain* |  |  |  |  |  | -2.495 | 0.754 | -3.310 | 0.0022 | ** |
| *Lung* |  |  |  |  |  | 5.193 | 0.891 | 5.828 | 0.0000 | **** |
| *Liver* |  |  |  |  |  | 4.112 | 0.932 | 4.414 | 0.0001 | **** |
| *Lung* |  |  |  |  |  | 4.546 | 0.756 | 6.015 | 0.0000 | **** |
|  | N = 40; df = 38; Adjusted R^2^ = 0.105; F-statistic = 5.545;  P-value = 0.0235 | | | | | N = 40; df = 34; Adjusted R^2^ = 0.7505; F-statistic = 24.46;  P-value = 0.0000 | | | | |

**Supplemental Table 6:** Multiple linear regression analysis. MLR was performed to test the relationship between score, count, and organ. For organ, GI tissue was selected as the reference tissue. Parameter is significant at α = 0.05; **< 0.005; **** < 0.00005; NS= not significant.
