# Supplementary material for: Plasmodium vivax Parasite Load Is Associated With Histopathology in Saimiri boliviensis With Findings Comparable to P vivax Pathogenesis in Humans
Source: Open Forum Infect Dis. 2019 Jan 19;6(3):ofz021. doi: 10.1093/ofid/ofz021 (PMC6436601; doi:10.1093/ofid/ofz021)
Supplement: ofz021_suppl_supplementary_table_7 [file ofz021_suppl_supplementary_table_7.docx]

| **Supplemental Table 7: Spearman’s Rank Coefficient Values** | | | | |
| --- | --- | --- | --- | --- |
| Variable 1 | Variable 2 | Spearman’s ρ | Adjusted P-value | Significance |
| Histopathological Score | Parasite Burden | 0.6034 | 0.0002 | *** |
|  | Maximum Parasitemia | 0.0768 | 1.0000 | NS |
|  | Days at Peak Parasitemia | -0.0037 | 1.0000 | NS |
|  | Proportion at Peak Parasitemia | 0.0683 | 1.0000 | NS |
|  | Parasitemia at Necropsy | 0.0163 | 1.0000 | NS |
|  | Duration of Infection | 0.0508 | 1.0000 | NS |
|  | | | | |

**Supplemental Table 7:** Spearman’s Rank coefficient test. Parasite tissue burden (parasite counts) and other parameters were tested to determine their association with pathology score. Spearman’s ρ is significantly not equal to 0 at α = 0.05, ***<0.0005, NS = not significant. All tests are two-sided. P-values adjusted for multiple comparisons with Bonferroni Correction.
