# Supplementary material for: Plasmodium vivax Parasite Load Is Associated With Histopathology in Saimiri boliviensis With Findings Comparable to P vivax Pathogenesis in Humans
Source: Open Forum Infect Dis. 2019 Jan 19;6(3):ofz021. doi: 10.1093/ofid/ofz021 (PMC6436601; doi:10.1093/ofid/ofz021)
Supplement: ofz021_suppl_supplementary_fig_1 [file ofz021_suppl_supplementary_fig_1.docx]

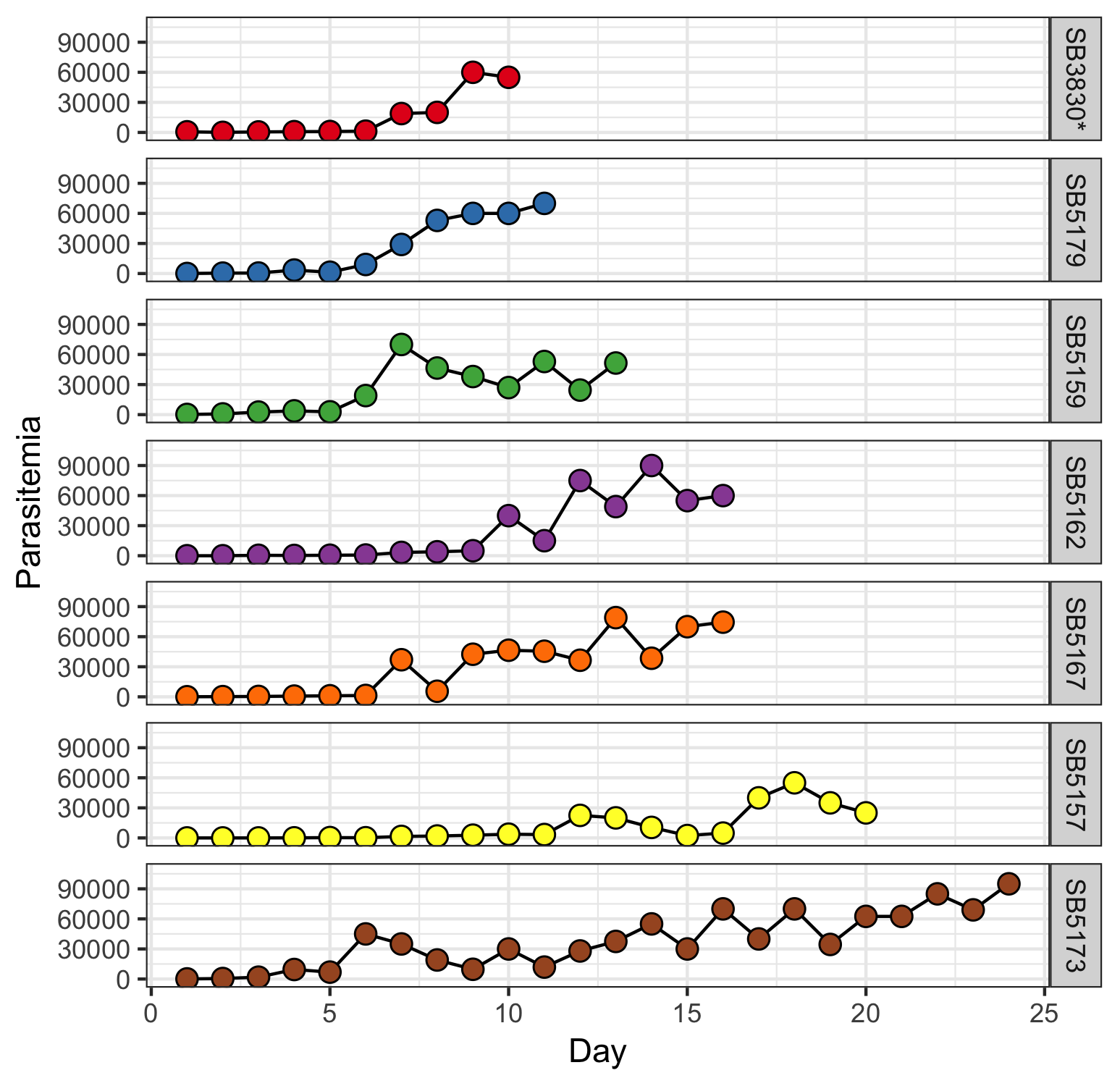


**Supplemental Figure 1:** Parasitemia kinetics from seven *S. boliviensis* monkeys infected sequentially with *P. vivax* iRBCs*.* One monkey (SB3830*) was spleen-intact. Parasitemias were recorded daily as parasites/μL, and the animals were sacrificed once they sustained parasitemias of at least 1% (approximately 50,000 parasites/μL). Monkeys were serially inoculated. Thawed cryopreserved iRBCs were initially inoculated into donor monkey, SB 5145 (not included in this cohort),_and on day 12, 1 ml of infected blood was transferred into SB 5162 and SB 5159. On day 10 of their infection, infected blood was transferred to SB 5167 from SB 5162, and to SB 5157 from SB 5159. On day 11 of the SB5157 infection, blood was transferred from this animal to SB 5157 and SB 5173. Finally, SB 3830 was inoculated with infected blood transferred from SB 5173 on day 7 of its infection.
